# Journalistic Note

**Published:** 1890-08

**Authors:** 


### Journalistic Note.

The *Western Medical Reporter*, in its July number, presents a most interesting one. The editor displays great good nature and equally good form in his reply to the criticisms in "Country Doctor."

